# Synovial cytokine expression in psoriatic arthritis and associations with lymphoid neogenesis and clinical features

**DOI:** 10.1186/ar3817

**Published:** 2012-04-27

**Authors:** Raquel Celis, Núria Planell, José L Fernández-Sueiro, Raimon Sanmartí, Julio Ramírez, Isidoro González-Álvaro, José L Pablos, Juan D Cañete

**Affiliations:** 1 Arthritis Unit, Rheumatology Department, Hospital Clinic de Barcelona and IDIBAPS, c/Villarroel, 170, 08036 Barcelona, Spain; 2 Plataforma de Bioinformática, Centro de Investigación Biomédica en Red de Enfermedades Hepáticas y Digestivas (CIBERehd), c/Córcega 180, 08036 Barcelona, Spain; 3 Rheumatology Division, Complejo Hospitalario Universitario, c/Xubias, 84, 15006 La Coruña, Spain; 4 Rheumatology Department, Hospital Universitario de la Princesa, IIS Princesa. c/Diego de León, 62, 28006 Madrid, Spain; 5 Instituto de Investigación, Hospital 12 de Octubre (I+12), Avda Córdoba s/n° 28041 Madrid, Spain

## Abstract

**Introduction:**

Psoriatic arthritis (PsA) is an autoantibody-negative immune-mediated disease in which synovial lymphoid neogenesis (LN) occurs. We determined whether LN is associated with specific patterns of inflammatory cytokine expression in paired synovial tissue (ST) and fluid (SF) samples and their potential correlation with the clinical characteristics of PsA.

**Methods:**

ST and paired SF samples were obtained from the inflamed knee of PsA patients. ST samples were immunostained with CD3 (T cell), CD20 (B cell), and MECA-79 (high endothelial vessels). Total ST mRNA was extracted, and the gene expression of 21 T-cell-derived and proinflammatory cytokines were measured with quantitative real-time PCR. SF concentrations of Th1, Th2, Th17, and proinflammatory cytokines were determined with the Quantibody Human Th17 Array. Clinical and biologic data were collected at inclusion and after a median of 27 months of follow-up.

**Results:**

Twenty (43.5%) of 46 patients had LN. Only two genes showed differences (Wilcoxon test, *P *< 0.06) in ST between LN-positive and LN-negative patients: interleukin-23A (*IL-23A*) (*P *= 0.058) and transforming growth factor-beta (*TGF-β1*) (*P *= 0.050). IL-23A expression was higher, and TGF-β1 expression was lower in LN-positive patients. ST IL-15 mRNA showed a nonsignificant trend toward higher expression in LN-positive patients, and SF IL-15 protein levels were significantly higher in LN-positive patients (*P *= 0.002). In all PsA patients, IL-23A mRNA expression correlated with C-reactive protein (CRP) (*r *= 0.471; *P *= 0.001) and swollen-joint count (SJC) (*r *= 0.350; *P *= 0.018), whereas SF levels of IL-6 and CC chemokine-ligand 20 (CCL-20) correlated with CRP levels (*r *= 0.377; *P *= 0.014 and *r *= 0.501; *P *< 0.0001, respectively).

**Conclusions:**

These findings suggest differences in the cytokine profile of PsA patients with LN, with a higher expression of IL-23A and IL-15 and a lower expression of TGF-β1. In the entire group of patients, IL-23 ST expression and CCL20 SF levels strongly correlated with markers of disease activity. This cytokine pattern was not accompanied by gross clinical or biologic differences between LN-positive and -negative patients. Taken together, these results suggest a role of the IL-17/IL-23 cytokine axis in synovial LN in PsA.

## Introduction

Psoriatic arthritis (PsA) is a chronic, inflammatory, musculoskeletal disease associated with skin psoriasis that is mediated by the immune system and can lead to significant bone and cartilage destruction, functional impairment, and reduced quality of life [1].

Histopathologic analyses of PsA versus rheumatoid arthritis (RA) synovial tissues have revealed differential characteristics that may be of potential value in the diagnostic classification of patients with undifferentiated arthritis, although they share basic features [2]. Of these, ectopic lymphoid neogenesis (LN) has been observed in similar proportions in patients with RA and PsA [3-5]. LN results from the organized aggregation of T and B cells around specialized vessels known as high endothelial venules (HEVs), and correlates with the ectopic expression of a restricted set of homing chemokines, which are physiologically involved in the traffic and tissue compartmentalization of T and B cells in secondary lymphoid organs [6].

A previous study of patients with PsA and LN by our group suggested that LN is a stable feature in these patients that is reversed only after therapeutic remission [3]. However, its pathogenetic and clinical significance in RA and PsA patients remains unclear.

LN in PsA patients remains intriguing because no specific autoantibodies have been described, and because rheumatoid factor (RF) and anti-citrullinated protein/peptide antibodies (ACPAs) are detected in low titers in only a small proportion of PsA patients [7]. However, B cells and LN structures could drive antibody-independent synovial inflammation through the development of specific T-cell responses or by enhancing cytokine production [8]. This might result in differences in the disease phenotype between patients with and without synovial LN, which might have prognostic interest.

To determine whether synovial LN is associated with differential patterns of cytokine expression, we quantified T-cell polarization (Th1/Th2; Th17/IL-23) and proinflammatory cytokines in paired synovial tissue (ST) and fluid (SF) samples of patients with PsA with and without synovial LN. We also analyzed the potential association between these mediators and markers of disease activity.

## Methods

### Patients and synovial tissues

Synovial biopsy specimens were obtained with needle arthroscopy from patients of Hospital Clinic (Barcelona, Spain) and Complejo Hospitalario Universitario, (La Coruña, Spain) meeting the CASPAR criteria for PsA [9], selected according to the presence of active knee synovitis (pain and inflammatory synovial fluid). Paired synovial fluid samples were also obtained at the same time from 42 of these patients. All patients gave informed consent, and the study was approved by the Ethics Committee of the Hospital Clinic of Barcelona, Barcelona, Spain.

Arthroscopy was performed under diagnostic and/or therapeutic (lavage) conditions with a 2.7-mm arthroscope (Storz, Tullingen, Germany). Eight synovial tissue samples were obtained from the suprapatellar pouch and the medial and lateral gutter in each patient [3]. Four samples were fixed in 4% formaldehyde and embedded in paraffin wax for immunohistochemistry, and the remaining four, collected on RLT lysis buffer (Qiagen, Crawley, West Sussex, UK for RNA extraction.

Patients were evaluated every 3 to 4 months, and the treatment was changed if the disease remained active (that is, DAS28 > 3.2 or ≥ 2 swollen and tender joints). Therapy with methotrexate was initiated (up to 20 mg/week if tolerated), and if no response occurred or adverse events were noted, patients were switched to anti-TNF-α or to combined therapy, according to their rheumatologist's judgment. Clinical and biologic data (joint pattern, DAS28, C-reactive protein (CRP) and erythrocyte sedimentation rate (ESR), disease-modifying antirheumatic drugs (DMARDs), and biologic therapy administered) were collected at study inclusion and the last clinical control. Psoriasis severity at inclusion was evaluated by Physician Global Assessment [10].

### Immunohistochemistry

Sequential sections of PsA synovial tissues were analyzed for the presence of lymphoid aggregates and the expression of the following markers by peroxidase immunohistochemical analysis. T cells were labeled with polyclonal rabbit anti-human CD3 (A0452; DAKO, Cambridge, UK); B cells, with mouse anti-human CD20 (clone L26, DAKO); and HEV, with rat anti-human PNAd (clone MECA-79; PharMingen, Oxford, UK). Antigen retrieval, which was required for most antibodies, was performed with microwave heating in 1 m*M *EDTA for 15 minutes.

Primary antibodies were developed with appropriate secondary biotinylated antibodies according to a biotin peroxidase-based method (ABC; Vector Laboratories, Burlingame, CA, USA), by using diaminobenzidine as chromogen. Sections were counterstained in Gill hematoxylin.

### Analysis of lymphoid aggregates

The highest grade of lymphoid aggregation within each synovial tissue sample was determined according to a previously described scoring method [11] based on the number of radial cell counts: grade 1, two to five radial cell counts; grade 2, six to 10 radial cell counts; and grade 3, more than 10 radial cell counts. The presence of T/B cell segregation and PNAd+ HEV within lymphoid aggregates was analyzed.

### Real-time quantitative PCR

Total RNA was extracted from ST samples of 46 PsA patients by using the RNeasy FFPE Kit (Qiagen) according to the manufacturer's recommendations. cDNA was synthesized by using a high-capacity cDNA archive kit (Applied Biosystems, Warrington, UK). The primers for *CCR7, IFN-γ, IL-1β, IL-2, IL-6, IL-7, IL-8, IL-10, IL-12A, IL-13, IL-15, IL-17A, IL-18, IL-21, IL-22, IL-23A, IL-28, IL-33, TGF-β1, TNF- α*, and lymphotoxin (*LTβ*), were purchased from Applied Biosystems, and gene expression was measured with duplicate quantitative real-time PCR by using the TaqMan Gene Expression Assay (Applied Biosystems).

The expression of 23 target genes and one housekeeping gene (*GAPDH*) was measured with qPCR. To quantify transcript levels, target-gene Ct values were normalized by using the DeltaCt method (ΔCt = Ct reference gene - Ct target gene). Fold changes were calculated by using the differences of medians of the two groups, and a nonparametric Mann-Whitney-Wilcoxon test was used to examine statistically different expression patterns between groups. A *P *value of ≤ 0.05 was considered statistically significant. Ct values were calculated by using the SDS 2.4 and RQ Manager software (Applied Biosystems).

### Quantification of cytokines in synovial fluid

SF cytokines were analyzed by using Quantibody Human TH17 Array 1 (GM-CSF, IL-1β, IL-2, IL-4, IL-5, IL-6, IL-10, IL-12p70, IL-13, IL-17A, IL-17F, IL-21, IL-22, IL-23, IFN-γ, CCL20, TGF-β1, TNF-α, and TNF-β) (RayBiotech, Norcross, GA, USA), according to the manufacturer's specifications. Each sample was prepared in quadruplicate. An Axon scanner 4000B with GenePix software was used to collect fluorescence intensities. Detection limits for cytokines are displayed on the manufacture's website [12].

IL-15 levels in synovial fluid supernatants were measured with enzyme-linked immunoassay (ELISA), as previously described [13].

### Statistical analysis

Numeric variables were described as median and interquartile range (IQR), and categoric variables, as frequencies and percentages. The Wilcoxon Rank Sum test or Kruskal-Wallis test was used to compare the distribution of numeric variables between groups.

The Fisher Exact test was used to compare categoric variables. Correlation between numeric variables was expressed by Spearman correlation coefficient, and the null hypothesis was tested (coefficient = zero).

Correlation between two categoric variables or between one numeric and one categoric variable was assessed by using the Fisher Exact test and the Wilcoxon Rank Sum test or Kruskal-Wallis test. Univariate and multivariate logistic regression models were performed to evaluate association with LN, and the area under the curve (AUC) of the receiver operating characteristic (ROC) curve was used to assess the performance of prediction models. Classifiers with an AUC > 0.5 have at least some ability to discriminate between groups. All analyses were performed by using R statistical environment, version 2.12.1 [14].

## Results

### Clinical and demographic data

Forty-six PsA patients (57% male), aged (median; IQR) 50 (37; 61) years were included; disease duration at inclusion was 7.5 years (3.7; 13), and follow-up time after inclusion was 27 months (9; 52). Twenty-three (50%) patients had oligoarthritis, 18 (39%), polyarthritis, and five (11%), mixed-pattern (peripheral arthritis with axial involvement. PGA was 21 (46%) clear, 22 (48%) mild, and three (6%) moderate.

C-reactive protein (CRP) was 0.7 (0.3; 2) mg/dl; TJC, 1 (1; 2); SJC, 1 (1; 2); and DAS28, 3.3 (3; 4.2). Three patients were RF positive at low titers (< 80 U/mL), and no patient was ACPA positive (> 25 U/mL) (Table 1).

**Table 1:** Clinical and biologic characteristics of patients stratified by lymphoid neogenesis

Demographic	All (*n *= 46)	LN+ (*n *= 20)	LN- (*n *= 26)	*P*
Age (years)	50 (37; 61)	48 (36; 59)	54 (38.5; 63)	NS
Male (*%*)	26 (57)	13 (65)	13 (50)	NS
Disease duration (years)	7.5 (3.75; 13)	6 (2; 14)	8 (4.5; 12.5)	NS
Follow-up (months)	27 (9; 52)	21 (8; 50)	27 (12; 55)	NS

Clinical status				

TJC	1 (1; 2)	1 (1; 2)	1 (1; 2)	NS
SJC	1 (1; 2)	1 (1; 2)	1 (1; 2)	NS
Articular pattern, *n *(%)				
Oligoarthritis	23 (50)	13 (57)	10 (43)	NS
Poliarthritis	18 (39)	7 (39)	11 (61)	
Mixed (peripheral plus axial)	5 (11)	2 (40)	3 (60)	
ESR (mm/h)	19 (10; 30)	20 (9; 31)	18 (12; 25)	NS
CRP (mg/dl)	0.72 (0.32; 2.01)	1.00 (0.51; 1.83)	0.44 (0.29; 2.32)	NS
DAS28	3.31 (3.01; 4.2)	3.31 (2.94; 4.14)	3.34 (3.01; 4.28)	NS
Erosive disease, *n *(%)	12 (26)	7 (35)	5 (19)	NS

Treatment				

DMARDs taken, *n *(*%*)	23 (50)	12 (60)	11 (42)	NS
TNF-blockers, *n *(*%*)	8 (17)	1 (5)	7 (27)	**0.041**

EULAR response	Treated (*n *= 28)	LN+ (*n *= 11)	LN- (*n *= 17)	*P*

No response, *n *(*%*)	8 (29)	3 (27)	5 (29)	0.541
Moderate, *n *(*%*)	7 (25)	4 (36)	3 (18)	
Good, *n *(*%*)	13 (46)	4 (36)	9 (53)	

Data are expressed as median (IQR) or as percentage.

Twenty-eight (60.3%) patients were treated during the follow-up: 23 with methotrexate, three of them combined with TNF-blockers, and five with TNF-blockers in monotherapy. In this cohort, the main target of treatment was arthritis. LN-negative patients were treated with significantly more TNF-blockers than were LN-positive patients, but this finding could reflect differences in the rheumatologist criteria (Table 1).

Twelve (26%) patients had erosive disease at the end of follow-up, without differences between LN-positive and -negative patients.

Of the 28 patients treated during follow-up, 71% achieved a EULAR response (46% good and 25% moderate), without differences between LN-positive and -negative patients (Table 1).

### Synovial cytokine expression in the entire group of PsA patients

All 21 cytokine genes studied were expressed in ST in most patients, although some were expressed in fewer than 50% of patients (*IL-13, IL-17A, IL-21, IL-22*, and *IL-28A*). In SF, IL-12 and IL-28A were not detected at all, and some cytokines were detected in only a small percentage of patients in comparison with mRNA expression. The exceptions were SF IL-6, IL-15, and CCL20, which were detected in either ST or SF in > 80% of patients (Tables 2 and 3). No correlation was found between the two compartments for any cytokine, probably because of the dissociation of cytokine expression between ST and SF.

**Table 2:** RT-PCR of 21 cytokine genes expressed as Delta Ct value and stratified by LN

	*n *(%) of positive patients	All *n *= 46	LN+ *n *= 20 (43.5%)	**LN**- ***n *= 26 (52.4%)**	Wilcoxon *P *value
*CCR7*	45 (98%)	8 (-10; -7)	-8 (-9; -7)	-8 (-10; -7)	0.465
*IFN-γ*	35 (76%)	-10 (-12; -9)	-10 (-11; -9)	-11 (-12; -8)	0.656
*IL-10*	45 (98%)	-7 (-8; -6)	-7 (-8; -7)	-7 (-8; -6)	0.312
*IL-12A*	41 (89%)	-9 (-10; -8)	-9 (-10; -8)	-10 (-11; -8)	0.188
*IL-13*	11 (24%)	-14 (-15; -13)	-15 (-16; -15)	-13 (-14; -11)	0.109
*IL-15*	42 (91%)	**-9 (-9; -8)**	**-8 (-9; -8)**	**-9 (-10; -8)**	**0.104**
*IL-17A*	15 (33%)	-11 (-13; -9)	-13 (-14; -11)	-11 (-11; -9)	0.121
*IL-18*	46 (100%)	-6 (-7; -6)	-7 (-7; -6)	-6 (-7; -5)	0.418
*IL-1β*	43 (94%)	-8 (-9; -7)	-9 (-9; -8)	-8 (-9; -7)	0.517
*IL-2*	29 (59%)	-12 (-14; -11)	-13 (-14; -11)	-12 (-14; -12)	0.846
*IL-21*	20 (43%)	-13 (-14; -12)	-13 (-14; -12)	-13 (-14; -12)	0.909
*IL-22*	7 (15%)	-12 (-14; -11)	-13 (-15; -12)	-11 (-13; -11)	0.629
*IL-23A*	45 (98%)	**-5 (-6; -5)**	**-5 (-6; -4)**	**-6 (-8; -5)**	**0.058**
*IL-28A*	21 (46%)	-10 (-12; -9)	-10 (-12; -8)	-11 (-11; -10)	0.622
*IL-33*	41 (89%)	-11 (-12; -9)	-11 (-12; -9)	-11 (-12; -9)	0.646
*IL-6*	44 (96%)	-8 (-10; -6)	-8 (-9; -7)	-8 (-10; -5)	0.706
*IL-7*	44 (96%)	-6 (-7; -6)	-6 (-7; -5)	-7 (-7; -6)	0.608
*IL-8*	46 (100%)	-4 (-6; -2)	-4 (-5; -3)	-3 (-6; -2)	0.877
*LTβ*	44 (96%)	-6 (-8; -5)	-6 (-7; -5)	-6 (-8; -6)	0.443
*TGF-β1*	46 (100%)	**-3 (-4; -2)**	**-3 (-4; -3)**	**-3 (-3; -2)**	**0.050**
*TNF-α*	46 (100%)	-7 (-8; -6)	-7 (-8; -6)	-7 (-8; -6)	0.816

**Table 3:** Cytokine levels (pg/mL) in patients with PsA stratified by LN

	*n *(%) of positive patients	All *n *= 42	LN+ *n *= 20 (47.6%)	LN- *n *= 22 (52.4%)	Wilcoxon *P *value
GM.CSF	4 (10%)	0 (0; 0)	0 (0; 0)	0 (0; 0)	0.051
IFN-γ	6 (14%)	0 (0; 0)	0 (0; 0)	0 (0; 0)	0.444
IL-1β	7 (7%)	0 (0; 0)	0 (0; 0)	0 (0; 0)	0.632
IL-2	21 (50%)	42 (0; 133)	0 (0; 151)	83 (0; 119)	0.851
IL-4	2 (2%)	0 (0; 0)	0 (0; 0)	0 (0; 0)	0.365
IL-5	19 (19%)	0 (0; 0)	0 (0; 0)	0 (0; 0)	0.440
IL-6	41 (98%)	1241 (503; 1,544)	1324 (764; 1,496)	1093 (258; 1,720)	0.462
IL-10	22 (52%)	4 (0; 13)	4.4 (0; 12)	0 (0; 14)	0.926
IL-12p70	0	0 (0; 0)	0 (0; 0)	0 (0; 0)	NA
IL-13	4 (10%)	0 (0; 0)	0 (0; 0)	0 (0; 0)	0.348
IL-15	38 (91%)	**114 (47; 197)**	**180 (109; 261)**	**62 (23; 115)**	**0.002**
IL-17	11 26%)	0 (0; 20)	0 (0; 7)	0 (0; 26)	0.757
IL-17F	12 (29%)	0 (0; 12)	0 (0; 6)	0 (0; 12)	0.728
IL-21	19 (45%)	0 (0; 817)	0 (0; 773)	0 (0; 958)	0.591
IL-22	1 (2%)	0 (0; 0)	0 (0; 0)	0 (0; 0)	0.317
IL-23	15 (36%)	0 (0; 212)	0 (0; 190)	0 (0; 262)	0.758
IL-28A	0	0 (0; 0)	0 (0; 0)	0 (0; 0)	NA
CCL20	34 (81%)	37 (6; 497)	23 (7; 284)	40 (1; 624)	0.752
TGF-β1	8 (19%)	0 (0; 0)	0 (0; 0)	0 (0; 446)	0.157
TNF-α	4 (10%)	0 (0; 0)	0 (0; 0)	0 (0; 0)	0.323
TNF-β	7 (17%)	0 (0; 0)	0 (0; 0)	0 (0; 0)	0.684

Data are expressed as median (IQR). NA, not applicable.

### Correlation of cytokines with markers of disease activity

IL-23 A mRNA expression was correlated with CRP (*r *= 0.471; *P *= 0.001) and SJC (*r *= 0.350; *P *= 0.018), whereas SF levels of IL-6 and CCL-20 correlated with CRP levels (*r *= 0.377; *P *= 0.014; and *r *= 0.501; *P *< 0.0001, respectively).

### Synovial cytokines and lymphoid neogenesis

**Figure 1:**
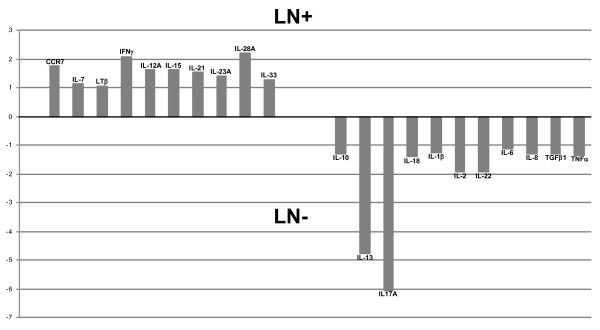
**Distribution of mRNA of all cytokines studied according their higher relative expression (fold change) as lymphoid neogenesis (LN) positive (**+**) or negative (-)**.

**Figure 2:**
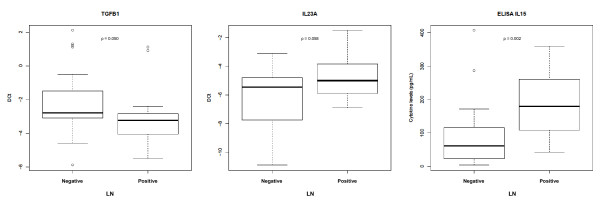
**Differential expression of TGF-β1 and IL-23A mRNA in synovial tissue, and IL-15 levels in synovial fluid, according to lymphoid neogenesis (LN) status**.

Twenty (43.5%) patients had synovial LN (follicular aggregates grade ≥ 2 with T/B cell segregation and HEV). Previous studies in RA showed a good correlation between LN and the expression of cytokines *IL-7 *and *LTβ *and several homing chemokines and their receptors [15]. In our PsA cohort, a modest, nonsignificant increase in CCR7, IL-7, and LTβ mRNA expression was detected in LN-positive patients (Table 2 and Figure 1). Differences between LN-positive and -negative patients (Wilcoxon *P *< 0.06) were found in ST in only two genes: *IL-23A *(*P *= 0.058) and *TGF-β1 *(*P *= 0.05). The expression of *IL-23A *was higher in LN-positive patients, and that of *TGF-β1 *was higher in LN-negative patients (Table 2 and Figure 2). The combination of *IL-23A *and *TGF-β1 *in a multivariate logistic regression model of factors influencing LN confirmed these genes as predictors of LN [AUC, 0.736; 95% CI, 0.5897 to 0.8823; *IL-23 A*, OR, 1.62 (1.06 to 2.48); *P *= 0.027; *TGF-β1*, OR, 0.72 (0.05 to 1.03), *P *= 0.075)].

However, TGF-β1 mRNA expression correlated with the number of B-cell grade 3 aggregates (*r *= 0.363; *P *= 0.046) and with the percentage of B cells in synovial samples (*r *= 0.508; *P *= 0.005). Although not significantly higher in LN-positive patients, IL-21 mRNA expression strongly correlated with the number of B-cell grade 3 aggregates (*r *= 0.686; *P *= 0.0048).

ST IL-15 mRNA was higher in LN-positive patients, with only a trend to significance (Wilcoxon *P *= 0.104) (Table 2). In SF, only IL-15 levels were significantly higher in LN-positive patients (Wilcoxon *P *= 0.0023; Table 2). Accordingly, SF IL-15 levels correctly classified patients as LN positive or negative (AUC, 0.791; 95% CI, 0.64 to 0.941; Table 3).

## Discussion

It has been suggested that ectopic LN might play a role in the pathogenesis of chronic inflammatory conditions by driving T-cell responses and producing proinflammatory cytokines [16]. The proportion of our PsA patients showing LN features is similar to that described previously and also to that of studies in RA [3,4]. The clinical significance of ectopic LN has recently been analyzed in RA, in which several studies point to more-refractory disease [4], greater inflammation [17], and more-erosive disease in this subset of patients [18].

Our study provides the first approach to this issue in PsA. Because PsA is not associated with B-cell responses, it represents a good model to explore whether T-cell polarization can be modified by LN, independent of B-cell responses. Our results show that PsA patients with synovial LN have a higher expression of *IL-23A *and a lower expression of *TGF-β1 *in synovial tissue compared with LN-negative patients. The combination of these two genes in a multivariable logistic regression model confirmed that they were associated with LN. Additional differences of lower magnitude in IL-15 and IL-21 mRNA expression also pointed to subtle differences in the cytokine profile of this PsA subset.

An unexpected finding of our study is the weaker correlation between molecular markers of LN, such as CCR7, IL-7, and LTβ, and LN, as defined by T-/B-cell aggregates, T/B compartmentalization, and MECA-79 epitope expression. The expression of these factors has been associated with synovial LN in RA patients and experimental animal models [6,19-21]. It is possible that the wide variation in the extension of lymphoid aggregates in different tissues, and the different histologic criteria used to identify LN may explain the variable associations of LN with molecular markers and disease severity found in different studies analyzing lymphocyte aggregation and organization [22]. This could lead to contradictory findings, as is the case with ST TGF-β1 mRNA expression, which was higher in LN-negative patients, but paradoxically, it correlated with B-cell aggregates grade 3 and strongly with the percentage of B cells in ST. Supporting this hypothesis, we specifically find a strong correlation between mature B-cell aggregates and IL-21 mRNA expression, but no significant differences according to LN classification.

We found an association between higher IL-23A mRNA expression and LN. Furthermore, IL-23A mRNA expression significantly correlated with SJC and CRP, and we noted a strong trend to correlate with DAS28. This finding did not correlate with the level of IL-23 in the SF, in which only a third of these patients had detectable IL-23.

IL-23 is expressed mainly by macrophages and dendritic cells and plays a key role in the differentiation and survival of Th17 cells and in IL-17 production by non-T cells [23]. IL-23 acts as an end-stage effector cytokine through direct action on macrophages [24], the main producers of proinflammatory cytokines in synovium. This mechanism of action is in line with our finding of a positive correlation between IL-23A ST expression and systemic markers of inflammation. However, a correlation between (serum) IL-23 levels and disease activity has previously been reported only in patients with RA [25,26].

The role of IL-23 in the pathogenesis of PsA is less clear. Genetic studies have confirmed the association of *IL-23A *and *IL-23R *genes with PsA susceptibility, underlining its potential pathogenetic relevance [27,28]. In addition, ustekinumab, an anti-IL-12/IL-23 antibody, has shown efficacy in PsA patients [29]. In ankylosing spondylitis, a disease grouped together with PsA in the concept of spondyloarthritis, a potential role for IL-23 has also been proposed [30]. Further research may better define the pathways through which IL-23 is produced and exerts its actions in PsA.

In this series, IL-15 mRNA was expressed in grossly 90% of paired ST and SF samples. Previous studies also detected IL-15 in PsA ST [31,32]. IL-15 stimulates the production of IL-17 in synovial monocytes and synergizes with IL-23 to increase IL-17 production. Additional effects on T- and B-cell survival and monocytes, mast cells, and neutrophils activation might also contribute to PsA pathogenesis [33,34]. In RA, IL-15 serum levels correlated with disease activity and may predict a severe disease course [35]. Our data show higher IL-15 expression in LN-positive patients, supporting its potential pathogenetic role. Overall, IL-15 and IL-23 expression would point to a link between LN and the Th17/IL-23 axis. However, the expression of Th17-related cytokines was moderate (IL-17A and IL-21) or very low (IL-22) in ST and SF, which may be in line with the low abundance of Th17 cells in PsA or SpA ST, in which the majority of IL-17-producing cells are non-T cells [36].

Regarding other factors potentially implicated in T-cell traffic and differentiation, in our study, SF CCL20 levels correlated with markers of inflammation. Previously a correlation was reported between SF CCL20 levels and the number of SF PMNs in PsA, but not with markers of disease activity [25]. CCL20 is a chemokine that attracts several types of cells, predominantly Th17 cells expressing the CCR6 receptor. In addition, it has been reported that IL-17F induces CCL20 production [37]. Upregulation of IL-23 and CCL20 in PsA supports the role of the IL-17/IL-23 axis disturbance in this disease. As expected, SF IL-6 levels correlated with CRP levels [38].

TGF-β1 is a multifaceted cytokine participating in many biologic processes: inflammation, fibrosis, lymphocyte recruitment, and lymphocyte-effector differentiation (Treg and Th17) [39]. Given that no previous reports exist of TGF-β1 in PsA, it is difficult to ascertain the clinical significance of the lower expression of these cytokines in LN in this disease.

Although these data suggest that synovial LN might be associated with a distinctive cytokine profile with a more-inflammatory phenotype, we found no clinical differences between LN-positive and -negative patients.

Our study has some limitations. First, all samples of ST and SF are from knee arthritis, which may not be representative of other inflamed joints in the same patient. However, previous studies in RA patients suggested that ST from an active joint is a good representation of other active joints in the same patient [40]. Another limitation is that this is a retrospective study, with established disease in the majority of patients, when the ideal scenario for evaluating the clinical significance of LN might be early untreated PsA patients [22]. Nevertheless, our exploratory study identifies some differences in the cytokine profile that support the existence of pathogenetic differences in the LN-positive PsA subset.

## Conclusion

The results of this study suggest differences in the cytokine profile of PsA patients with LN, with a higher expression of *IL-23A *and IL-15 and a lower expression of *TGF-β1*. This cytokine pattern was not accompanied by gross clinical or biologic differences between LN-positive and -negative patients. In the entire group of patients, *IL-23A *ST expression and CCL20 SF levels strongly correlated with markers of disease activity. Taken together, these results suggest a role of the IL-17/IL-23 axis in synovial LN in PsA.

## Abbreviations

CCL20: CC-Chemokine Ligand 20; CCR7: CC-Chemokine Receptor type 7; CRP: C-reactive protein; DAS28: 28-joint Disease Activity Score; DMARDs: disease-modifying antirheumatic drugs; ESR: erythrocyte sedimentation rate; IFN-γ: interferon gamma; IL: interleukin; LT-β: lymphotoxin β; TGF-β1: transforming growth factor β1; TJC: tender-joint count; SJC: swollen-joint count; TNF-α: tumor necrosis factor-α; TNF-β: tumor necrosis factor-β.

## Competing interests

The authors declare that they have no competing interests.

## Authors' contributions

JDC had full access to all the data in the study and takes responsibility for the integrity of the data and the accuracy of data analysis. JDC and JLP were responsible for the study design. RC, NP, JLFS, RS, JR, IGA, JLP, and JDC performed data acquisition, analysis, interpretation, and final approval of the manuscript. NP performed statistical analysis. Manuscript preparation was by JDC, JLP, and RC. All authors read and approved the final manuscript.
